# Learning from developing countries in strengthening health systems: an evaluation of personal and professional impact among global health volunteers at Addis Ababa University’s Tikur Anbessa Specialized Hospital (Ethiopia)

**DOI:** 10.1186/s12992-014-0064-x

**Published:** 2014-09-05

**Authors:** Heidi Busse, Ephrem A Aboneh, Girma Tefera

**Affiliations:** Department of Surgery, University of Wisconsin School of Medicine and Public Health, 600 Highland Avenue, Madison, WI 53792 USA; Addis Ababa University, College of Health Sciences, Addis Ababa, Ethiopia

**Keywords:** Global health, Twinning partnership, Collaboration, Health systems partnership, Reverse innovation, Africa

## Abstract

**Background:**

The positive impact of global health activities by volunteers from the United States in low-and middle-income countries has been recognized. Most existing global health partnerships evaluate what knowledge, ideas, and activities the US institution transferred to the low- or middle-income country. However, what this fails to capture are what kinds of change happen to US-based partners due to engagement in global health partnerships, both at the individual and institutional levels. “Reverse innovation” is the term that is used in global health literature to describe this type of impact. The objectives of this study were to identify what kinds of impact global partnerships have on health volunteers from developed countries, advance this emerging body of knowledge, and improve understanding of methods and indicators for assessing reverse innovation.

**Methods:**

The study population consisted of 80 US, Canada, and South Africa-based health care professionals who volunteered at Tikur Anbessa Specialized Hospital in Ethiopia. Surveys were web-based and included multiple choice and open-ended questions to assess global health competencies. The data were analyzed using IBRM SPSS® version 21 for quantitative analysis; the open-ended responses were coded using constant comparative analysis to identify themes.

**Results:**

Of the 80 volunteers, 63 responded (79 percent response rate). Fifty-two percent of the respondents were male, and over 60 percent were 40 years of age and older. Eighty-three percent reported they accomplished their trip objectives, 95 percent would participate in future activities and 96 percent would recommend participation to other colleagues. Eighty-nine percent reported personal impact and 73 percent reported change on their professional development. Previous global health experience, multiple prior trips, and the desire for career advancement were associated with positive impact on professional development.

**Conclusion:**

Professionally and personally meaningful learning happens often during global health outreach. Understanding this impact has important policy, economic, and programmatic implications. With the aid of improved monitoring and evaluation frameworks, the simple act of attempting to measure “reverse innovation” may represent a shift in how global health partnerships are perceived, drawing attention to the two-way learning and benefits that occur and improving effectiveness in global health partnership spending.

## Introduction

Although the effectiveness of global health spending has been questioned [1–4], global health initiatives have mobilized vast financial and human resources to address complex public health issues in low- and middle-income countries. Global health funding in the form of development assistance for health (DAH) grew from $5.6 billion USD in 1990 to almost $21.8 billion USD in 2007 [5]. In the US alone, government funding for global health programs grew from $1.7 billion USD in FY2001 to $8.9 billion USD in FY 2012 [6]. Additionally, private foundations, academic/research institutions, and the corporate sector contribute to DAH and have an increasing role in shaping international health policies and programs [4,7,8], making it difficult to neatly capture all of the individual and private sector contributions to global health initiatives [5,9]. Despite the generosity of contributions toward solving global health problems, these initiatives have at times been criticized for weakening health systems because they may require that host countries establish new coordination structures, limit the authority or participation of existing leadership, minimize local stakeholder engagement, ignore cultural values, and fail to strengthen communication and trust among members [10].

Current evaluation indicators for global health partnerships tend to focus on the knowledge, ideas, and activities the institution from the developed country transferred to the developing country. Even if a partnership was developed with expectations of reciprocal relationships, shared accountability, and equity, implicit in this traditional global health paradigm is the assumption that change and knowledge are solely transferred from developed to developing countries. Despite so many resources invested in global health, the impact of this spending on individuals and institutions from developed countries has neither been well assessed nor reported. This type of impact has been referred to as “reverse innovation” [11] in global health literature, a term borrowed from the business sector. Reverse innovation refers to an innovation first seen or applied in a developing country before being adapted to an industrialized setting. General Electric (GE) successfully implemented this approach in its design of lower-cost, portable ultrasound machines, initially designed for lower-resource settings [12]. Between 2002 and 2011, global sales of portable ultrasound machines rose from $5 million USD to $280 million USD, with an average annual growth rate of 50 percent [12]. Their resulting technological innovation brought the company profit but also helped health providers deliver improved services to health care institutions otherwise unable to afford the standard machine. Reverse innovation as an organizational approach can not only lead to profitability and “success in developing countries… [but also serve as a] prerequisite for continued vitality in developed ones” for businesses that wanted to compete in global markets [12]. The concept of reverse innovation – that innovations developed within emerging countries can be extended to other settings – may have application to health care and other sectors where there is interaction between and flow of people, resources, and ideas across regions, as new ways of doing things are needed in order to solve our shared global challenges [13].

The three objectives of this study are to identify what kinds of personal, professional and institutional impact global health collaborations have on US-based partners involved with a twinning partnership between Addis Ababa University and the University of Wisconsin-Madison, advance this emerging body of knowledge, and improve understanding of what kinds of measures can be used to measure “reverse innovation” in global health.

## Background

The global health challenges of today are complex in nature and consume vast resources. Thus, they require an interdisciplinary approach that considers whole systems rather than individual problems. One strategy being promoted to advance aid effectiveness and improve health impact is to use collaboration to build effective global health partnerships [14]. The importance of partnership has been described in several international documents, including the Paris Declaration on Aid Effectiveness, the Accra Agenda for Action, and the Busan Partnership for Effective Development Cooperation. The outcomes of partnerships – increased effectiveness, efficiency, engagement and ownership – should be realized by all stakeholders, both those providing and receiving funding.

Brought together by a shared concern about the challenges in delivering emergency medical services in Ethiopia, Addis Ababa University’s Tikur Anbessa Specialized Hospital (AAU/TASH) and the University of Wisconsin School of Medicine and Public Health (UW), both academic medical institutions, formed a global health partnership with People To People (a diaspora network of Ethiopian health professionals) in 2009 based on a 6-phase twinning partnership model [15]. The partnership’s goal was to enhance and strengthen adult and pediatric emergency care at AAU/TASH by building institutional and human resource capacity and increasing the number of medical professionals trained to deliver emergency care. The Ethiopia Emergency Medicine (EM) partnership followed the twinning model to address the entire spectrum of emergency services. The core principles of a twinning partnership are:

1. Community involvement and volunteerism,
2. Broad-based institutional relationships,
3. Peer-to-peer collaborative relationships,
4. Professional exchanges and mentoring,
5. Joint stakeholder involvement and empowerment, and
6. Local political support

The Ethiopia EM twinning partnership engaged volunteers from within the UW, diaspora networks, and academic institutions from across the US, Canada, and South Africa. Volunteers were recruited by the UW but selected by both AAU and UW team members together. Selection criteria included: at least five years of clinical experience (for clinical volunteers), at least five years of experience teaching residents or nurses (for teaching volunteers), degree in their respective profession (*e.g.,* MD, RN, or MPH), prior experience working in limited resource settings, effective communication and teamwork skills, and a desire to serve in another country. Neither prior global health experience nor previous work in Ethiopia was mandatory. The majority of the global health volunteers participated in 1- or 2-week trips to deliver educational trainings, provide clinical mentoring, and assist in graduate level program development at AAU/TASH.

## Methods

The study population included health care professionals who had traveled to Ethiopia between 2009–2013 as volunteers with the AAU/UW twinning partnership. The sampling frame contained email addresses of 80 professionals who had travelled to Ethiopia in order to learn what kinds of change they attributed to their twinning program participation. The survey questions were modeled upon the seven core competencies of global health developed by the Association of Schools of Public Health. Closed and open-ended questions were developed that addressed personal and professional impact specific to this partnership. These seven core competencies include:

1. Capacity strengthening
2. Collaboration and partnerships
3. Ethical reasoning and professionalism
4. Health equity and social justice leadership
5. Program management
6. Cultural awareness
7. Strategic analysis and evaluation

The survey instrument was tested prior to fielding using a small sample (n = 5) of professionals having similar experiences to those of the final participants. Once testing was complete, the final survey was administered using Qualtrics®, a web-based survey hosting program. This study was considered a program evaluation and granted approval as an exemption from the Institutional Review Board of the University of Wisconsin-Madison. Data were analyzed using IBM SPSS® version 21 for quantitative analysis, and the open-ended responses were coded using constant comparative analysis to identify key themes for qualitative analysis. The qualitative data were coded into two deductive categories to align with our study question: personal impact and professional impact. Data reflecting personal impact were coded within categories into themes and nodes within themes by two members of the project team trained in qualitative research methods. The frequency of the emergent themes was identified. Among data coded for personal impact, six themes were indicated by 10% or more of respondents. Among data coded for professional impact, eight themes were indicated by 10% or more of respondents. Results from the quantitative analyses are presented as frequencies and percentages. Measures of association between categorical variables were determined using the Chi-square test at 95 percent confidence level.

## Results

The questionnaire for the survey was sent out to all 80 volunteers and 63 (79 percent) responded. The 17 non-responders were similar in age, gender, and profession to the responders. Fifty-two percent of the participants were male and over 60 percent were 40 years of age and older. Fifty-seven percent of the participants traveled to Ethiopia one time only, while 15 percent had traveled four or more times. Table 1 reports the respondent characteristics. More than half of respondents (62 percent) were physicians, while 30 percent were nurses. Eighty-seven percent of the respondents reported that they provide clinical care in specialties that included anesthesia, emergency medicine, family medicine, internal medicine, nursing, obstetrics/gynecology, pediatrics, radiology, and surgery.

**Table 1:** **Profile of Survey Respondents (n = 63)**

Male	52%
Female	48%
20-29 years	13%
30-39 years	23%
40-49 years	38%
50+ years	26%
MD	62%
BSN/RN	30%
MPH	15%
Student/Other	13%
PhD	7%
1 Trip to AAU/TASH	57%
2 or 3 Trips	28%
4+ Trips	15%
Prior global health experience	65%
No prior experience	35%

Participation in the Ethiopia twinning partnership was the first global health experience for 35 percent of respondents. Among the 65 percent who had had prior experience, 60 percent had made 5 or fewer previous trips, 20 percent had made 6–10 previous trips, and 20 percent had made 11 or more previous trips.

The most frequently cited reasons for wanting to participate in an exchange trip with the Ethiopia twinning partnership were a desire to share skills/knowledge with others (82 percent), enjoy teaching others (79 percent), interest in mission/service work (74 percent), and desire to learn about another country’s health system (61 percent). The least cited reason was to advance one’s career/professional development (39 percent).

### Personal impact

Among all respondents, 83 percent reported they accomplished their trip objectives, 95 percent would participate in future activities and 96 percent would recommend participation to other colleagues to participate. When participants were asked to compare what goals they hoped to accomplish BEFORE going to Ethiopia with what they believed to have accomplished AFTER returning from Ethiopia, there was a marked difference. Pre-departure goals emphasized the transfer of knowledge/skills, teaching, and working to change systems. In contrast, the goals and achievements they believed to be most important after returning from Ethiopia were self-reflective and emphasized how the respondents’ were changed by the experience. These accomplishments include learning from their Ethiopian counterparts, building relationships, experiencing/appreciating a different culture, and approaching their profession differently. Eighty-nine percent of respondents reported personal impact as a result of participation in the twinning partnership program. The types of personal impact varied, and a summary of the most frequently reported themes that emerged from the open-ended responses are found in Table 2: collaboration and teamwork, cultural experience/sharing, fulfillment/appreciation for selected profession, global connectedness, gratitude, and serving others.

**Table 2:** **Ways in which volunteers were impacted PERSONALLY that they attribute to the global health experience at Tikur Anbessa Specialized Hospital**

**Collaboration and teamwork**	Broadened understanding of challenges in managing complex systems, and the importance for local ownership of problems and solutions.
**Cultural experience and awareness**	Heightened awareness of the difficulties in working in environments where you do not speak the language. Learned about and gained a respect for Ethiopia and the Ethiopian people’s ability overcome adversity. Learned to let go of personal and cultural expectations.
**Fulfillment and appreciation for selected profession**	Inspired renewed enthusiasm to personal & professional goals and (re)affirmed commitment to work in global health.
**Global connectedness**	Increased awareness that global health is our problem. Changed a person’s worldview of the US health system compared to other countries, particularly the inequitable allocation of resources. Made aware of all the work we have yet to do to create a more just and sustainable world and ensure everyone has access to health resources.
**Gratitude**	Gained deeper appreciation and gratitude, especially for their chosen profession and the ability to make a difference in people’s lives.
**Serving and training others**	Changed approach and learned new skills for teaching/mentoring of medical students, residents, and nursing students. Developed a personal interest in being a part of advancing medical care and health systems in Ethiopia.

### Professional impact

Seventy-three percent reported impact on their professional development that they attributed to participation in this global health partnership. Respondents were asked to check boxes (they were allowed to check more than one) to indicate what kinds of professional impact they had experienced, and also given space to write open-ended responses. The types of professional development most frequently checked were an expanded professional network (73 percent), skills in planning and implementing workshops, (73 percent), and contribution to their curriculum vitae (70 percent). Table 3 summarizes the qualitative responses collected. Previous global health experience, multiple prior trips, and the desire for career advancement were all associated with positive impact on professional development at p < 0.05.

**Table 3:** **Ways in which volunteers were impacted PROFESSIONALLY that they attribute to the global health experience at Tikur Anbessa Specialized Hospital**

**Clinical skills development**	Provided with first-hand experience of new pathologies not previously witnessed other than in textbooks. Improved skills in communicating with patients and colleagues/team members. Changed approach to patient examinations.
**Improve quality of patient care and delivery of health services**	Forced to think about health disparities that exist and how patients in the US access the health system, and ways to reduce barriers (particularly those related to language and cultural differences).
**Increased volunteerism**	Gained an opportunity to provide clinical, academic, and research training/services to others. Renewed interest in volunteering more frequently, both globally and locally.
**Leadership**	Added to professional development, including academic outputs, recognition from supervisor/chair, and promotion. Asked to participate on professional committees, international in scope.
**Program management**	Developed skills in designing and planning workshops. Re-learned basic skills that had been forgotten working in a resource-rich environment, such as process improvement, change management, and leadership.
**Relationship building**	Expanded professional network. Learned about Ethiopian cultural practices in health delivery, such as end-of-life and post-mortem care.
**Resource utilization**	Reduced resource consumption of disposable resources at work. Changed frequency/approach to ordering diagnostic lab studies and imaging studies. Reconsidered excessive use of and reliance on technology in the US health system.
**Teaching skills**	Improved skills in curriculum development. Changed approach in teaching medical students, residents, and departmental staff.

### Clinical impact

#### Clinical impact

More nurses reported impact on their clinical practice than physicians, 75 percent and 38 percent, respectively (p < 0.001). Among all volunteers with a clinical practice, the skill most frequently cited as being enhanced by this partnership was improved pedagogical skills for teaching (57 percent), followed by communication with patients (52 percent), improving systems within one’s department (43 percent), and improved skills for conducting patient assessments (43 percent).

### Framework for measuring personal and professional impact

The AAU/UW twinning partnership impacted US-based volunteers at the personal and professional levels. Survey results indicate volunteers experienced change in all seven of the global health competencies developed by the Association of Schools of Public Health: capacity strengthening, collaboration and partnerships, ethical reasoning and professionalism, health equity and social justice leadership, program management, cultural awareness, and strategic analysis and evaluation [16]. Using this as a model for the survey design, the UW developed a framework to present the ways in which professional impact may be manifested among global health volunteers (Table 4).

**Table 4:** **Framework for professional impact experienced by UW global health volunteers: Health system competencies and examples of how they may be demonstrated**

**Competency 1. Capacity Strengthening**	- Coordinate and/or manage diverse teams
	- Design health worker trainings
	- Monitor and evaluate health worker trainings
**Competency 2. Collaboration and Partnerships**	- Build trust with colleagues
	- Ensure health partnerships represent diverse perspectives
	- Set goals and expectations for health partnerships
**Competency 3. Ethical Reasoning and Professionalism**	- Analyze ethical issues that impact diverse cultures/backgrounds
	- Promote integrity in professional practice
	- Hold self and colleagues accountable to practice standards
**Competency 4. Health Equity and Social Justice Leadership**	- Assess disparities in the distribution of health resources
	- Empower vulnerable populations to make decisions that support health/well-being and are culturally appropriate
	- Advocate for social justice principles in patient care and/or institutional/hospital policies
**Competency 5. Program Management**	- Conduct a formative assessment for program planning that considers local stakeholders’ resources/input
	- Apply scientific evidence throughout program planning, implementation, and evaluation
	- Utilize program evaluation results to inform modifications/improvements
**Competency 6. Cultural Awareness**	- Describe how culture influences health decisions and outcomes
	- Design health advocacy strategies that consider diverse cultural, socio-economic, religious, and other backgrounds
	- Analyze factors that influence public health
**Competency 7. Strategic Analysis and Evaluation**	- Implement a community health needs assessment
	- Identify relationships between social determinants of health and health outcomes in a local context
	- Propose strategies for improving health systems in limited resource settings

## Discussion

Partnerships in global health are means for stakeholders with shared goals to achieve more collectively than they could have achieved acting independently. However, the term “partnership” has different meanings in different contexts. One framework suggested by Rosenberg *et al.* [17] is that global health partnerships fall along a spectrum, from coordination to cooperation to close collaboration. A partnership focused on coordination has a clear purpose (e.g., mobilizing aid for natural disasters), is often time-bound, and is coordinated by a primary authority figure/institution. At the opposite end of the spectrum, the authors found that “close collaboration” partnerships form in response to complex, long-term situations that present social and political challenges and require integrated teams willing to invest time and expertise. The type of partnership needed changes depending upon the context and ultimate goals, and subsequently results in different outcomes. Thus, it is important to consider what kind of partnership exists to know what kinds of impact are realistic to expect.

Along that spectrum, the AAU/UW twinning partnership falls nearer to close collaboration. Defining characteristics of the AAU/UW twinning partnership include:

1. Shared commitment to a single, complex health issue that required an interdisciplinary approach.
2. Core team of individuals worked together over time.
3. Partners brought institutional resources dedicated to the partnership.
4. Regular, bi-directional exchanges to foster two-way learning and sharing.
5. Collective accountability for setting, monitoring, and accomplishing goals.
6. Participation was primarily volunteer-based.
7. Timeframe was measured in years, not months.

The AAU/UW partnership initially included a monitoring and evaluation (M&E) framework with targets, indicators, data sources, and individual/institutional responsibility for reporting in order to ensure accountability to funders. This framework was based upon goals and objectives outlined in the shared work plan and complied with reporting guidelines; however, the indicators were focused on collecting data to monitor changes seen in Ethiopia. What this M&E model failed to capture was what kinds of change and impact the partnership had on the US partners. Even though the partnership was developed in the spirit of collaboration, reciprocal relationships, and equity, the structure of the M&E framework had an implicit assumption that change is one-directional. The work plan indicators focused on how the partner institution from the developed country would transfer knowledge, ideas, and innovation in emergency health systems to the developing country. The AAU/UW partnership developed the new M&E framework (Table 4) using the seven global health competencies to help assess what ideas, knowledge, and innovation the Ethiopian partners taught the US partners, and what kinds of reverse innovation could be attributed to the global health partnership. This emphasis on mutuality of benefits between partners – which Nigel Crisp [18] termed “co-development” – reflects a shift toward a partnership framework that values interdependence, transparency, and accountability [19].

Additionally, one of the defining characteristics of the partnership was its emphasis on bi-directional exchanges. These bi-directional exchanges consisted of teams composed of diverse members (e.g., medical school administration, physicians, nurses, program coordinators, and residents) who were selected based on the specific trip goals. For example, the exchange trips included leadership development and capacity building, technical training, and work plan development, and delivery of content happened in both Ethiopia and Wisconsin. This fostered two-way learning and sharing, an innovative model that has demonstrated impact in other partnerships [20]. Knowing what kinds of impact can result from global health partnerships can improve the monitoring and evaluation methods. Further, it helps demonstrate how global service can be part of organizations’ strategic plans to create cultures of excellence that improve staff performance and the financial performance of an organization, giving managers and administrators the information to know what kind of institutional benefits could result from supporting faculty, staff, and student participation in global health activities.

## Limitations

Although the current study provides important insights into the nature of reverse learning that can occur with global health partnerships, our findings should be viewed in light of several limitations. First, the sample size for the study was relatively small and findings may not necessarily be generalizable to other settings. Within this study sample, the initial selection criteria included prior experience of working in limited resource settings. However, more than half (57 percent) of the volunteers only traveled to Ethiopia one time, for 35 percent this was their first global health experience and this compared with only 15 percent who had traveled more than four times to Ethiopia. The subsequent impact that this has on the findings is an area of further inquiry to understand its effect on the generalizability of results. Second, as described in the Discussion section, the AAU/P2P/UW twinning partnership functioned as a close collaboration that emphasized long-term relationships, bi-directional exchanges, and strong diaspora leadership. Thus, these findings may be unique to this model of partnership and may not be applicable to partnerships that do not possess similar structures. Further, the partnership engaged two academic medical institutions (i.e., AAU/TASH and the UW), leading to a unique combination of institutional resources, staff, and support that were able to support this collaboration. Finally, the study was initially conceptualized as a program evaluation and thus findings were initially intended to inform program improvements.

## Conclusion

Involvement in partnerships has been shown to play an important part in increasing intangible assets for individuals, organizations and society. Because professionally and personally meaningful learning happens often during global health outreach activities, investment in partnerships can be crucially important and have public value in the form of problem solving, enhanced competence, leadership skills, and innovation. However, more understanding is needed about what this impact is and what conditions are required to create effective learning environments to quantify the impact of this spending. With the aid of improved monitoring and evaluation frameworks such as the global health competencies matrix the UW developed, the simple act of attempting to measure “reverse innovation” may represent a shift in how global health partnerships are perceived, drawing attention to the two-way learning and benefits that occur and contributing to improved effectiveness in global health partnership spending.

## References

[1] Spicer N, Aleshkina J, Biesma R, Brugha R, Caceres C, Chilundo B, Chkhatarashvili K, Harmer A, Miege P, Murzalieva G, Ndubani P, Rukhadze N, Semigina T, Walsh A, Walt G, Zhang X (2010). National and subnational HIV/AIDS coordination: are global health initiatives closing the gap between intent and practice?. Global Health.

[2] Hancock G (1989). Lords of Poverty: The Power, Prestige, and Corruption of the International Aid Business.

[3] Moyo D (2009). Dead Aid: Why Aid is Not Working and How There is Another Way for Africa.

[4] Easterly W (2006). The White Man’s Burden: Why the West’s Efforts to Aid the Rest Have Done So Much Ill and So Little Good.

[5] Ravishankar N, Gubbins P, Cooley RJ, Leach-Kemon K, Michaud CM, Jamison DT, Murray CJ (2009). Financing of global health: tracking development assistance for health from 1990 to 2007. Lancet.

[6] Salaam-Blyther T: *U.S. Global Health Assistance: Background and Issues for the 113*^*th*^*Congress.* Washington, DC: Congressional Research Service Report for Congress; 2013:7–5700.

[7] Kanavos D, Rudisill C, Hockley T (2007). Critical review of the IFPMA Health Partnerships Survey.

[8] MacArthur T (2006). The scaling up of private philanthropy: implications for development outcomes.

[9] McCoy D, Chand S, Sridhar D (2009). Global health funding: how much, where it comes from and where it goes. Health Pol Plann.

[10] Busse H, Azazh A, Tefera G, Teklu S, Tupesis JP, Woldetsadik A, Wubben RJ, Tefera G (2013). Creating change through collaboration: A twinning partnership to strengthen emergency medicine at Addis Ababa University/Tikur Anbessa Specialized Hospital. Acad Emerg Med.

[11] Syed SB, Dadwal V, Rutter P, Storr J, Hightower JD, Gooden R, Carlet J, Bagheri Nejad S, Kelley ET, Donaldson L, Pittet D (2012). Developed-developing country partnerships: benefits to developed countries?. Global Health.

[12] Immelt JR, Govindarajan V, Trimble C (2009). How GE Is Disrupting Itself. Harv Bus Rev.

[13] Govindarajan V (2012). A Reverse-Innovation Playbook: Insights from a company that developed products for emerging markets and then brought them back home. Harv Bus Rev.

[14] US Global Health Initiative (2013). Promoting Partnerships to Advance Global Health Initiative Objectives.

[15] American International Health Alliance (2010). AIHA Twinning Partnership Toolkit.

[16] Association of Schools of Public Health (2011). Global Health Competency Model.

[17] Rosenberg ML, Hayes ES (2010). McIntyre MH, Neill N: *Real Collaboration: What it Takes for Global Health to Succeed*.

[18] Crisp N (2010). Turning the world upside down: the search for global health in the twenty-first century.

[19] Boydell L (2007). The benefits of partnerships publications.

[20] Syed SB, Dadwal V, Storr J, Riley P, Rutter P, Hightower JD, Gooden R, Kelley E, Pittet D (2013). Strengthening the evidence-policy interface for patient safety: enhancing global health through hospital partnerships. Global Health.

